# Efficacy of cytotoxic agents used in the treatment of testicular germ cell tumours under normoxic and hypoxic conditions *in vitro*

**DOI:** 10.1038/sj.bjc.6601375

**Published:** 2003-11-25

**Authors:** S Koch, F Mayer, F Honecker, M Schittenhelm, C Bokemeyer

**Affiliations:** 1 Department of Oncology, Hematology, Immunology, and Rheumatology, Medizinische Klinik, University of Tübingen Medical Center, Otfried-Müller-Str. 10, Tübingen 72076, Germany

**Keywords:** testicular germ cell tumours (TGCTs), cytotoxic drugs, normoxia, hypoxia, *in vitro*

## Abstract

Platinum-based chemotherapy is the main treatment element to achieve cure for patients with metastatic germ cell tumours. Drug resistance in testicular germ cell tumours (TGCTs) is rare and the reasons are not fully understood. While recent investigations have indicated decreased efficacy of chemotherapy in several tumour types under hypoxic conditions, this aspect has not been investigated in TGCTs so far. Furthermore, for cisplatin – the most active drug in this disease – controversial effects of hypoxia on cytotoxic efficacy have been reported. The relative efficacy of cytotoxic agents for the treatment of TGCT patients was studied in three different cell lines derived from human embryonal carcinomas (EC) in an *in vitro* hypoxia model. NT2, 2102 EP, and NCCIT were tested for their sensitivity towards cisplatin, etoposide, bleomycin, 4-OOH-ifosfamide, carboplatin, paclitaxel, gemcitabine, oxaliplatin, irinotecan, and mitomycin C under normoxic and hypoxic conditions using the MTT assay. Inhibitory concentrations IC_50_ of the tested agents under both conditions were compared. Selected results were confirmed by flow-cytometric assessment of the apoptotic index. In all cells, doubling times were prolonged in hypoxia (NT2<NCCIT<2102 EP). All drugs were less effective under hypoxic conditions, including mitomycin C (eg, 1.6-fold increase of IC_50_ in hypoxia compared to normoxia for NT2) and cisplatin (eg, NT2: two-fold increase). The relative effect of hypoxia on the IC_50_ depended mainly on the cell line, and to a lesser extent on the drug. The results indicate that the reduced cell proliferation in hypoxia might be an important factor, but not the only determinant of a reduced cytotoxicity. In view of the broad spectrum of drugs with different modes of action tested, the relative resistance cannot be mediated by substance-specific resistance mechanisms like hypoxia-induced upregulation of P-glycoprotein or increased DNA-repair capacity, since many unrelated drugs were affected to a comparable extent in their efficacy by hypoxia. This study also provides the rationale to test the hypothesis whether improving tumour oxygenation by raising haemoglobin concentrations, for example, with erythropoietin in patients with TGCTs receiving chemotherapy may improve the outcome.

Testicular germ cell tumours (TGCTs) are highly sensitive to cisplatin-based combination chemotherapy, and most patients with this disease can be cured today. Nevertheless, 10–15% of patients with metastatic disease will not achieve a disease-free survival with currently available treatment strategies, and finally die of their disease. The reasons for intrinsic or subsequently developed treatment resistance in these patients have not yet been fully explored (Mayer *et al*, 2003). A reduced intratumoral oxygen tension (hypoxia) has been reported in a variety of malignant tumours (Vaupel and Hoeckel, 1998; Semenza, 2000), and may limit the effectiveness of cytotoxic drugs. A positive correlation between the intracellular oxygen tension (pO_2_) and the efficacy of a radiotherapy has been described as early as 1931 (Mottram, 1931). These experimental data are endorsed by more recent clinical findings in patients with cancer of the uterine cervix, and head and neck tumours undergoing radiotherapy (Hoeckel *et al*, 1996; Nordsmark *et al*, 1996). The intratumoral oxygen tension depends at least partly on the haemoglobin level of the blood (Vaupel and Hoeckel, 1998; Becker *et al*, 2000).

Under hypoxic conditions, proteins like the vascular endothelial growth factor (VEGF) and the hypoxia-inducible factor 1 alpha (HIF-1*α* are upregulated in cancer cells (Zhong *et al*, 1999; Kondo *et al*, 2000; Cooke *et al*, 2001; López-Barneo *et al*, 2001). Induction of antiapoptotic proteins like Bcl-2 or of the multidrug resistance gene (MDR1) product P-glycoprotein is associated with HIF-1*α* overexpression, and may lead to resistance against chemotherapeutic agents (Goldstein, 1996; Zhong *et al*, 1999; Kinoshita *et al*, 2001; Comerford *et al*, 2002). The loss of apoptotic mechanisms (deprivation of p53) and loss of DNA mismatch repair (MMR) in hypoxia render cells both hypersensitive to acquire microsatellite instability and to the development of drug resistance (Lin *et al*, 2000, 2001; Kondo *et al*, 2001).

Most cytotoxic agents show a positive relation between oxygen tension and efficacy in cell culture. For mitomycin C, higher efficacy in hypoxia has been reported (Kennedy *et al*, 1983; Luk *et al*, 1990; Yamagata *et al*, 1992; Sanna and Rofstad, 1994). For cisplatin, the most active drug for TGCTs, the results reported are contradictory, but seem to indicate an enhanced drug activity under hypoxic conditions in various models (Liang, 1996; Skov *et al*, 1998; Kovacs *et al*, 1999). Other cytotoxic drugs with described clinical activity in refractory germ cell tumours, such as paclitaxel, gemcitabine and oxaliplatin, have not yet been tested for their activity under hypoxia *in vitro*. In addition, both the choice of the specific chemotherapeutic agent as well as the tumour type may influence the relative impact of hypoxia in the treatment setting.

Erythropoietin (epoietin) offers the chance to effectively ameliorate anaemia in cancer patients receiving chemotherapy (Cella *et al*, 2003). Next to a proven benefit regarding quality of life, epoietin might potentially affect the efficacy of the anticancer treatment used by raising the *p*O_2_ in tumour tissues. In order to provide the preclinical rationale for a clinical study with chemotherapy and epoietin in patients suffering from GCTs, we have investigated the *in vitro* efficiency of cytotoxic agents with different modes of action such as alkylating agents (eg, ifosfamide), platin derivatives, antibiotics (mitomycin C, bleomycin), gemcitabine, topoisomerase I (irinotecan) and II (etoposide) inhibitors, and the taxane derivative paclitaxel under normoxic and hypoxic conditions in established TGCT cell lines. Furthermore, the model system chosen here is used to discuss the relative efficacy of different cytotoxic agents in relation to the most effective drug cisplatin.

## MATERIALS AND METHODS

### Anticancer drugs

The drugs used were: cisplatin (CDDP; Bristol-Myers Squibb, München, Germany), oxaliplatin (Sanofi-Synthelabo GmbH, Berlin, Germany), carboplatin (Bristol-Myers Squibb), gemcitabine (Lilly Deutschland, Bad Homburg, Germany), etopophos (etoposide phosphate, VP-16; Bristol-Myers Squibb), bleomycin (Mack, Illertissen, Germany), mitomycin C (medac, Wedel, Germany), irinotecan (Aventis Pharma, Frankfurt/M., Germany), and 4-hydroperoxyifosfamide (4-OOH-ifosfamide; Asta Medica, Frankfurt/M., Germany). These agents were dissolved in *distilled water.* The semisynthetic agent paclitaxel (Sigma, Deisenhofen, Germany) from *Taxus baccata* was dissolved in DMSO (Sigma) and used without exceeding a final concentration of DMSO 0.1% (v/v), which by itself is not a toxic concentration for the cell lines studied.

### TGCT cell lines and culture conditions

Three established TGCT cell lines derived from human embryonal carcinomas were tested for their sensitivity towards different chemotherapeutic agents. The TGCT cell line NTera-2 (NT2/D1, a cell line known to be able to differentiate into neurons); ATCC CRL-1973 used in this study was maintained in DMEM with 4.5 g l^−1^ glucose and stable glutamine (Invitrogen, Karlsruhe, Germany), the 2102 EP cell line (Wang *et al*, 1981) and NCCIT (ATCC CRL-2073) were cultured in DMEM/F-12 with 2 mM L-glutamine (Biochrom). All cell lines were grown with the addition of 10% fetal calf serum (FCS; Biochrom, Berlin, Germany) and 1% penicillin/streptomycin (Biochrom) at 37°C in a humid atmosphere containing 5% CO_2_ as monolayers in 75 cm^2^ cell culture flasks.

### Cell proliferation in normoxia *vs* hypoxia

For the assessment of doubling times, the cells were cultured in normoxic (20% O_2_) and hypoxic (continuous flow of 0.1 l min^−1^ of a mixture of 94% N_2_, 5% CO_2_, and 1% O_2_) conditions. Briefly, individual cells were spread out in six-well plates and viable cells were counted in their logarithmic growth phase after 48 and 70 h to calculate the population-doubling times under both conditions by trypane blue (0.4%; Sigma) exclusion.

For determination of cell cycle progression, NT2 and NCCIT cells were grown in 25 cm^2^ culture flasks in normoxia and hypoxia for 48 h. Further processing was performed according to the method of Nicoletti *et al* (1991). In brief, the supernatant and adherent cells were harvested, washed, and suspended in 0.5 ml hypotonic lysis buffer (0.1% sodium citrate, 0.1% Triton X-100) containing 25 *μ*l of a 1 mg ml^−1^ propidium iodide (PI) stock solution (50 *μ*g ml^−1^ final concentration). Analysis of the cell cycle phase was performed by flow cytometry on a FACScalibur (Becton Dickinson, Heidelberg, Germany), using the CellQuest analysis software.

### Determination of pH value of the medium for untreated cells

NT2 and NCCIT cells were cultured with 10 ml complete medium in 25 cm^2^ cell culture flasks under normoxic and hypoxic conditions. After 72 h, the pH of the cell medium was measured using the pH meter model pH330 (WTW, Weilheim, Germany) and compared with 37°C annealed normoxic and hypoxic medium without cells.

### *In vitro* drug-sensitivity assay

The MTT assay was performed as previously described (Sieuwerts *et al*, 1995). In brief, the cell lines NT2, 2102 EP, and NCCIT were rinsed with phosphate-buffered saline (PBS, Biochrom), trypsinised and resuspended in 1 ml of the appropriate culture medium, to count the cells in a haemacytometer chamber. In all, 4 × 10^3^ cells/well were seeded in 96-well plates to ensure their logarithmic growth. Cells were allowed to adhere over night, serial dilutions of the chemotherapeutic agents were added to octuplicate wells at concentrations from 1 nM to 0.1 mM. The cells were exposed to the drugs for additional 72 h under normoxic and hypoxic conditions. Additionally, NT2 and NCCIT cells were treated with mitomycin C for 72 h under hypoxic conditions using culture medium adjusted to pH 6.5.

After this, the drug-containing medium was removed and 0.2 ml MTT solution (final concentration: 0.5 mg/mL MTT; Sigma) was added in ther medium. The plates were incubated for 2 h and then the medium was removed, 0.1 ml DMSO was added, the plates agitated for 15 min and the optical density read using a photometer (MRX Revelation, Dynex Technologies, VWR International, Bruchsal, Germany) at 570 nm.

All experiments were replicated separately twice or more if the values of increase in IC_50_ in hypoxia compared to normoxia were greater than 20%, to ensure reproducibility. The results are expressed as drug concentrations that inhibit cell growth by 50% (inhibitory concentration; IC_50_). The IC_50_ of the tested agents under both conditions were estimated graphically from the dose–response curves and compared. The relative increase in IC_50_ in normoxia *vs* hypoxia was assessed.

### Induction and quantification of apoptotic cells

In all, 1 × 10^5^ cells/well for normoxia and 2 × 10^5^–4 × 10^5^ cells/well for hypoxia were seeded in six-well plates. After overnight preincubation, serial dilutions of cisplatin and paclitaxel were added to the medium in chosen concentrations for NT2 and 2102 EP cells. Annexin-V labelling of the cells was performed as recommended by the manufacturer (Roche Diagnostics; Mannheim, Germany). In brief, after 72 h floating, adherent cells were harvested using trypsine-EDTA solution after PBS washing. The cell suspension was spun down and the cell pellet was resuspended in 0.1 ml of a marker solution (2 *μ*l Annexin-V-Fluos (50 × concentrated; Roche) in HEPES buffer (10 mM HEPES, 140 mM NaCl, 5 mM CaCl_2_; pH 7.4) containing 2 *μ*l of a 50 *μ*g ml^−1^ PI stock solution). The suspension was incubated for 15 min in the dark; afterwards, 0.2 ml HEPES buffer were added and kept on ice until further processing. Analysis of cell size and fluorescence intensity was performed flow cytometrically on the FACScalibur. After exclusion of necrotic debris, apoptotic and nonapoptotic (viable) cells were assessed.

## RESULTS

### Drug sensitivity in normoxic conditions and relative effect of hypoxia

The drug sensitivity was assessed by the MTT assay under normoxic (20% O_2_) and hypoxic (1% O_2_) conditions for 72 h. The results are summarised in Table 1

**Table 1:** Mean values of IC_50_ in normoxia and hypoxia (±standard deviation) of cytotoxic drugs after 72 h in culture of embryonal carcinoma (EC)-derived cell lines (NT2, 2102 EP, and NCCIT)

	**NT2**			**2102 EP**			**NCCIT**		
	**Average**			**Average**			**Average**		
**Agent**	**Normoxia IC_50_ (±s.d.)**	**Hypoxia IC_50_ (±s.d.)**	**Relative increase in IC_50_ (H : N) (±s.d.)**	**Normoxia IC_50_ (±s.d.)**	**Hypoxia IC_50_ (±s.d.)**	**Relative increase in IC_50_ (H : N) (±s.d.)**	**Normoxia IC_50_ (±s.d.)**	**Hypoxia IC_50_ (±s.d.)**	**Relative increase in IC_50_ (H : N) (±s.d.)**
Cisplatin	0.42 (±0.12)	0.83 (±0.25)	2 (±0)	0.8 (±0.04)	4.1 (±0.42)	5 (±0.28)	1.7 (±0.59)	12 (±3.2)	8 (±1.2)
Oxaliplatin	1.4 (±0.23)	3.8 (±0.15)	2.9 (±0.55)	1.3 (±0.09)	47 (±0.28)	35 (±2.6)	2.6 (±1.39)	62 (±25)	26 (±7.7)
Carboplatin	2.95 (±0.48)	14 (±4.04)	4.7 (±0.55)	6 (±2.55)	>100 (±n.d.)	>12 (±>3.5)	12 (±1.84)	>100 (±n.d.)	>8 (±>1.1)
Gemcitabine	0.055 (±0.044)	0.09 (±0.08)	1.6 (±0.08)	0.53 (±0.08)	>100 (±n.d.)	>100 (±n.d.)	0.9 (±0.09)	>100 (±n.d.)	>100 (±n.d.)
Etopophos	0.16 (±0.1)	0.18 (±0.1)	1.1 (±0.07)	0.2 (±0.15)	>100 (±n.d.)	>100 (±n.d.)	0.36 (±0.06)	>100 (±n.d.)	>100 (±n.d.)
Bleomycin	0.11 (±0.014)	2.1 (±0.39)	19 (±1.1)	0.16 (±0)	>75 (±>35)	>100 (±n.d.)	1.1 (±0.07)	>100 (±n.d.)	>95 (±>7)
Mitomycin C	0.04 (±0.014)	0.06 (±0.01	1.6 (±0.21)	0.4 (±0)	9 (±0.92)	23 (±2.3)	0.59 (±0.06)	7 (±0.21)	12 (±1.5)
Irinotecan	0.43 (±0.082)	0.74 (±0.08)	1.7 (±0.38)	1.3 (±0.08)	>100 (±n.d.)	>79 (±>2.1)	1.8 (±0.49)	44 (±9)	25 (±1.6)
Ifosfamide	2.85 (±1.2)	3.6 (±1.3)	1.3 (±0.11)	5.2 (±1.1)	80 (±23)	15 (±1.1)	7 (±1.1)	34 (±6)	4.7 (±0.04)
Paclitaxel	0.0035 (±6.08 E-4)	0.004 (±7.4 E-4)	1.1 (±0.07)	0.0038 (±1.53 E-4)	>83 (±>29)	>100 (±n.d.)	0.0043 (±7.1 E-5)	88 (±0.71)	>100 (±n.d.)

Additionally, the relative increase (±s.d.) of IC_50_ in hypoxia compared to normoxia (H : N) is listed. Values in *μ*M. N – normoxia. H – hypoxia. s.d. – standard deviation. n.d. – not defined.

.

Under normoxic conditions, the sensitivity towards cisplatin of the different cell lines varied by factor 4 at the IC_50_ values. Carboplatin showed the least cytotoxicity on an equimolar basis of all drugs tested in normoxia. In contrast, for oxaliplatin, the IC_50_-values varied only by a factor 2 in the three EC-derived cell lines. Apart from cisplatin, paclitaxel showed the highest activity in the three cell lines. No correlation was found between the sensitivity to paclitaxel and that to cisplatin under normoxic conditions.

**Figure 1:**
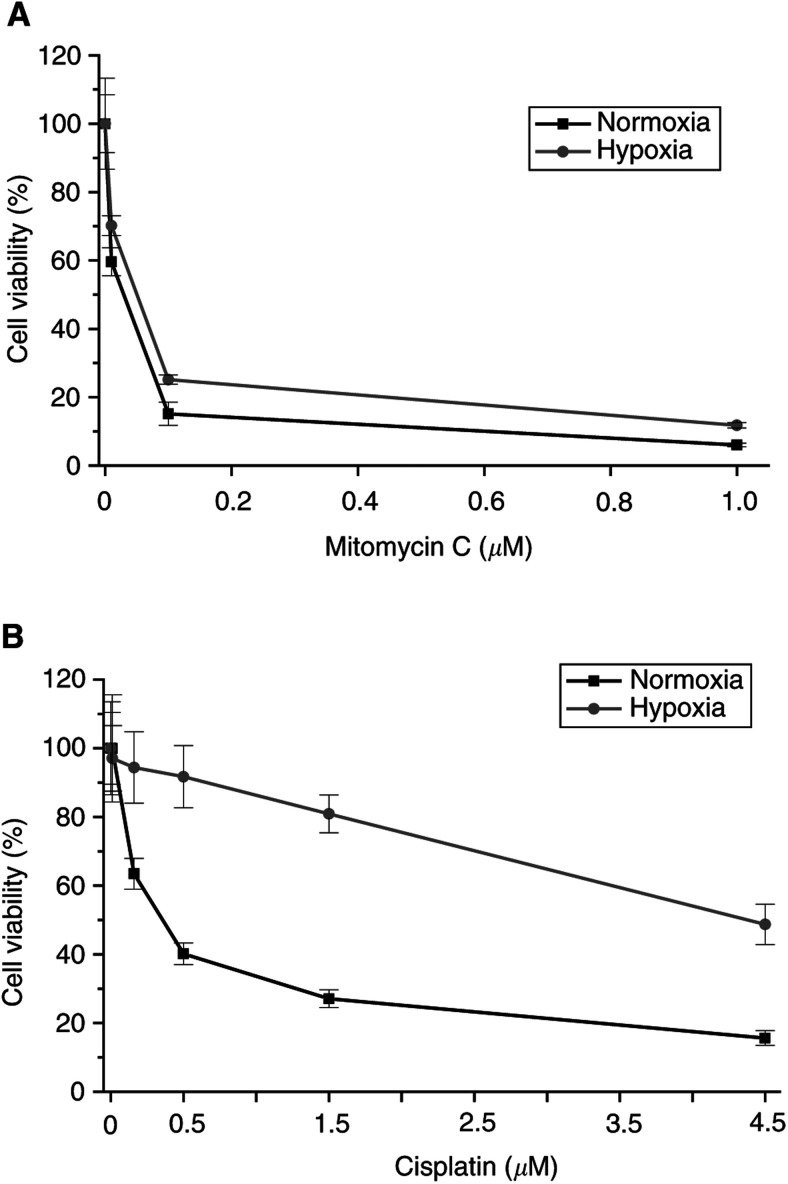
Cell viability, expressed as percent of the control (%means±s.d., which is indicated by the bars of the line plots) of EC cells in culture after 72 h. (**A**) NT2 cells treated with mitomycin C. (**B**) 2102 EP cells treated with cisplatin. Note that both drugs, mitomycin C and cisplatin, are less effective in hypoxia.

Under hypoxic conditions, all drugs tested were less effective (Table 1), including mitomycin C (eg, increase of IC_50_ in normoxia compared to hypoxia for NT2: 1.7-fold increase, Figure 1A

, and 2102 EP: 25-fold increase) and cisplatin (eg, NT2: two-fold increase, and 2102 EP: five-fold increase, Figure 1B). For mitomycin C, experimental modification of the extracellular pH to 6.5 did not result in an enhanced activity in NT2 and NCCIT cells during hypoxia (data not shown).

**Figure 2:**
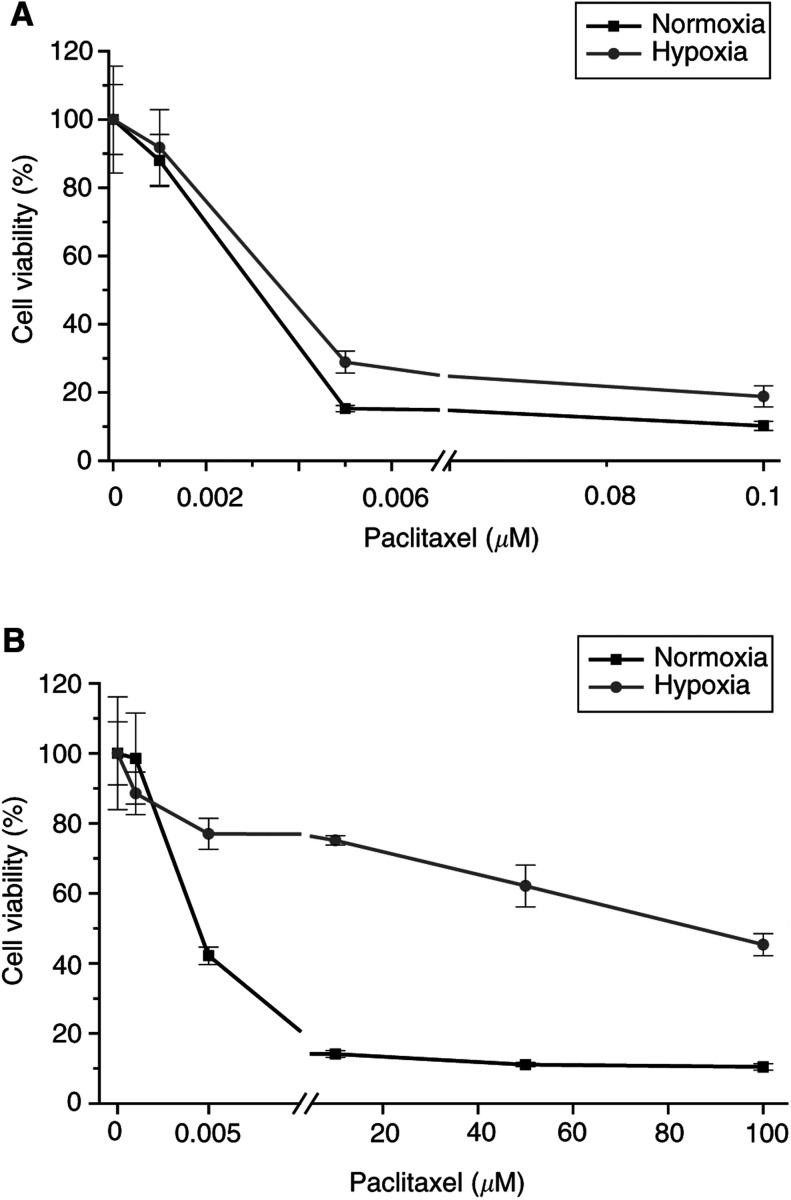
Cell viability, expressed as percent of the control (%means±s.d., which is indicated by the bars of the line plots) of EC cells in culture after 72 h treated with paclitaxel. (**A**) NT2 cells. (**B**) NCCIT cells.

The relative effect of hypoxia on the IC_50_ depended strongly on the cell line. NT2 cells showed a minor effect in chemosensitivity in hypoxia *vs* normoxia (eg, etopophos: 1.1-fold increase), 2102 EP cells exhibited overall a stronger effect of hypoxia (eg, etopophos: >100-fold increase). Additionally, the effect depended only to a restricted extent on the drug, but more clearly on the cell line used (eg, NT2/paclitaxel: 1.2-fold increase with an IC_50_ in normoxia: 3.1 nM, and hypoxia: 3.7 nM; Figure 2A

, and NCCIT/paclitaxel: >100-fold increase with an IC_50_ in normoxia: 4.3 nM, and hypoxia: 87 *μ*M; Figure 2B).

### Cell proliferation in normoxia *vs* hypoxia

**Figure 3:**
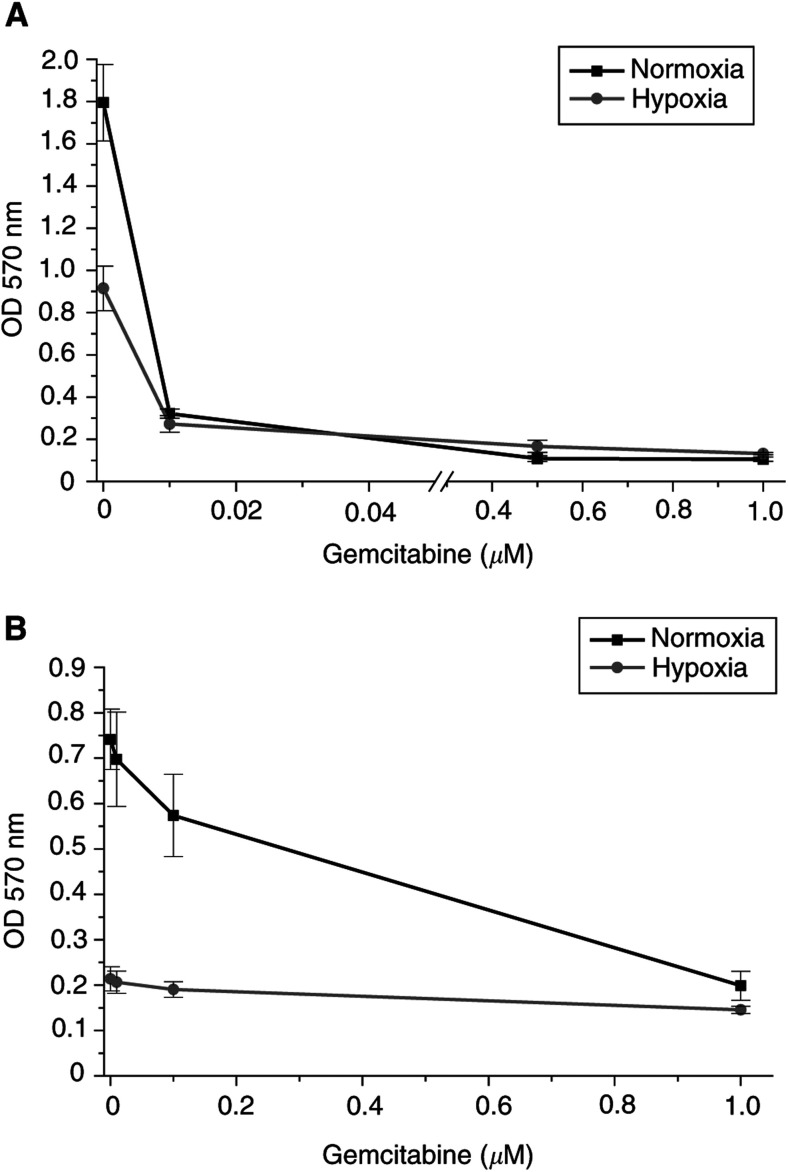
Cell growth, expressed as optical density at 570 nm (ODmeans±s.d., which is indicated by the bars of the line plots) of EC cells in culture treated with gemcitabine after 72 h. (**A**) NT2 cells. (**B**) 2102 EP cells. Note the reduced cell growth rate in hypoxia, which did not correlate with drug resistance. OD – optical density. nm – nanometers.

In normoxia, doubling times of the three cell lines were 23, 25, and 35 h for NCCIT, NT2, and 2102 EP, respectively. Under hypoxic conditions, NT2 cells showed a slower cell growth requiring 36 h for cell doubling. 2102 EP and NCCIT stopped growing under hypoxic conditions. In 2102 EP, the cell number dropped by approximately 51% and in NCCIT by 4%. The reduced growth rate was correlated with chemosensitivity of the TGCT cell lines under hypoxic conditions *in vitro* (see Table 1). However, it did not strictly correlate with the relative resistance to all drugs. For example, 2102 EP cells treated with gemcitabine (Figure 3B

) displayed an average increase of IC_50_ in hypoxia >100-fold compared to normoxia and five-fold for cisplatin (see Figure 1B). Compared to 2102 EP cells, NCCIT cells showed an improved survival in hypoxia, but they also indicated an average increase of IC_50_ in hypoxia compared to normoxia for gemcitabine >100-fold, and eight-fold for cisplatin. In contrast, NT2 treated with gemcitabine displayed a similar cell expansion under normoxic and hypoxic conditions (Figure 3A), with an average increase of 1.2-fold.

**Figure 4:**
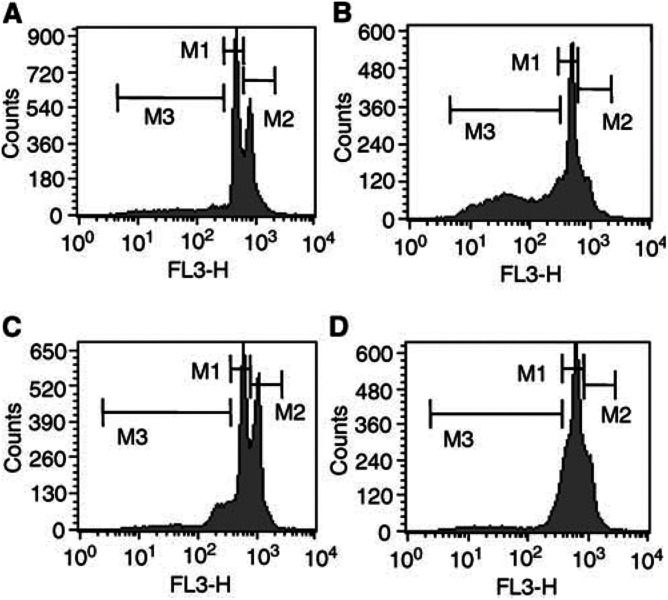
Histogram plots of the cell cycle analysis of NT2 (**A**) and NCCIT cells (**C**) in normoxia and NT2 (**B**) and NCCIT (**D**) in hypoxia after 48 h by flow-cytometric staining with PI. M1 – G1 phase. M2 – G2 phase. M3 – apoptotic cells.

Flow-cytometric analysis of the cell cycle progression revealed that hypoxic conditions induced a G1 arrest for NT2 and NCCIT cells (Figure 4B and D

) after 48 h, while under normoxic conditions no cell cycle phase synchronisation occurred (Figure 4A and C). Additionally, Figure 4B shows apoptosis (leakage of fragmented DNA from apoptotic nuclei; fraction M3) induced by hypoxia in NT2 cells compared to the cell line NCCIT (Figure 4D).

After 72 h in normoxia, the pH of the medium dropped from pH 7.8 to 6.7 for NT2 and from pH 7.6 to 6.4 for NCCIT. In hypoxia, the pH of the medium dropped only from pH 7.7 to 7.6 for NT2 and from pH 7.5 to 7.3 for NCCIT in the same time span.

### Induction of apoptosis

To evaluate the achieved differences of drug susceptibility of the TGCT cells in normoxia and hypoxia, the results from the colorimetric MTT assay were verified by flow cytometry. Viable cells (exclusion of PI and Annexin) and cells killed by cisplatin and paclitaxel (exclusion of PI, binding of Annexin) after a 72 h drug exposure of NT2 and 2102 EP were analysed by quantitating PI/Annexin-V labelling. The flow-cytometric results confirmed that cisplatin and paclitaxel are more effective in normoxia. For cisplatin, the relative resistance increased two-fold as measured by MTT, and 3.6-fold as assessed by FACS in NT2 cells in hypoxia compared to normoxia.

## DISCUSSION

In this *in vitro* study, three different TGCT cell lines were used to investigate the efficacy of several cytotoxic agents. The cell lines differed in their relative sensitivity to cisplatin by factor of 4. For oxaliplatin, the activity was almost similar in NT2 (cisplatin-sensitive) and 2102 EP (cisplatin-resistant) cells, and increased only by a factor 2 in NCCIT (cisplatin-resistant) cells. These *in vitro* data corroborate our previous clinical data describing a palliative oxaliplatin-based treatment option in patients with cisplatin-refractory germ cell cancer (Kollmannsberger *et al*, 2002). Carboplatin showed cross-resistance to cisplatin and a markedly lower activity on an equimolar level. Among the various agents used in this study, paclitaxel was very active in all cells, with no relative increase in IC_50_ values in cells where cisplatin was clearly less active. In line with this finding, Motzer *et al* (1995) described a marked efficacy of this drug in a teratocarcinoma cell line, particularly in cisplatin-resistant cells.

The main objective of this *in vitro* study was to investigate the relative efficacy of several chemotherapeutic agents used in the treatment for metastatic TGCTs during normoxic and hypoxic conditions. The oxygen content used in hypoxia models ranges from <0.1 to 1%. Culturing of the different GCT-derived cell lines in an atmosphere containing 1% oxygen induced a growth arrest and, in case of NT2 and 2102 EP cells, also cell deaths. Therefore, lowering the oxygen content further would have precluded a meaningful analysis due to lack of viable cells. To our knowledge, there are no data on the physiologic oxygen content in primary TGCTs or in metastases. Frequently encountered widespread necrotic areas suggest an insufficient blood supply and consequently hypoxia at least in some areas of these tumours.

Hypoxia has been shown to induce resistance against various agents and radiation (Brown and Giaccia, 1998; Hoeckel and Vaupel, 2001; Koukourakis *et al*, 2001; Vaupel *et al*, 2001). Conflicting data have been described for cisplatin, the most active drug in the treatment of TGCTs (Liang, 1996; Skov *et al*, 1998; Kovacs *et al*, 1999). The impact of hypoxia on the efficacy of the chemotherapeutic agents cisplatin, etoposide, bleomycin, ifosfamide, and carboplatin, all used in standard chemotherapy regimens for TGCTs, and of paclitaxel, gemcitabine, and oxaliplatin, drugs now used in patients with cisplatin-refractory disease (Bokemeyer *et al*, 1996; Motzer *et al*, 2000; Einhorn, 2002; Kollmannsberger *et al*, 2002; Shelley *et al*, 2002) was studied in three different cell lines. All drugs were less effective under hypoxic conditions. In contrast to data obtained from other tumour entities (Kennedy *et al*, 1983; Luk *et al*, 1990; Yamagata *et al*, 1992; Sanna and Rofstad, 1994; Liang, 1996; Skov *et al*, 1998; Kovacs *et al*, 1999), this also held true for the use of cisplatin and mitomycin C in GCTs.

Particularly for mitomycin C, this finding is unexpected. Mitomycin C has been postulated to be an alkylating agent requiring reduction for activity, and anaerobic conditions enhance the cytotoxicity (Iyer and Szybalski, 1964). Rockwell (1986) showed that the cytotoxic effects of mitomycin C increased at acidic pH culture conditions *in vitro*. At a low pH (6.0–7.0), mitomycin C can be spontaneously reduced to an alkylating species without enzymatic activation, while, in the physiologic pH range (7.0–7.4), the cytotoxic effect of mitomycin C does not vary with the pH (Rockwell, 1986). In our system, the pH of the medium of untreated cells under hypoxia was in the physiologic range and did not change after 72 h, probably due to the slower growth of NT2 cells or the growth arrest of NCCIT cells in hypoxia. Compared to that, the pH of the medium of untreated normoxic cells decreased to acidic pH values between 6.0 and 7.0. However, mitomycin C was also less effective in hypoxia at an experimentally acidified pH (6.5). Furthermore, this study demonstrates that mitomycin C already exhibited a significant cytotoxic effect in TGCT cells during hypoxia with 1% *p*O_2_ *in vitro*. In contrast to these results, Kennedy *et al* (1980) and Teicher *et al* (1981) achieved a selective toxicity of mitomycin C in mouse mammary tumour cells using considerable lower (<0.1%) oxygen tensions prior to the addition of the drug. As mitomycin C acts in a cell cycle-dependent manner, the pronounced effect of hypoxia on proliferation and the observed G1/S arrest might prevail the bioreductive activation in our model.

These findings may also serve as a rationale for clinical studies on tumour oxygenation and response to chemotherapy in GCT patients. A previous retrospective analysis of haemoglobin values at the end of treatment and prognosis in GCT patients undergoing sequential dose intensive chemotherapy has indicated that patients with a haemoglobin level <10.5 g dl^−1^ postchemotherapy may have a significantly inferior outcome (Bokemeyer *et al*, 2002). Tumour oxygenation depends, among other factors, on the haemoglobin content of the blood. Hence, correction of tumour-associated anaemia – for example, with recombinant erythropoietin – may improve the *p*O_2_ in tumour tissue. The use of erythropoietin in anaemic cancer patients has been studied to reduce the need for transfusions and to improve the quality of life (QOL). In patients with head and neck tumours receiving erythropoietin, an improved outcome of treatment has also been suggested. Based on the results presented, the hypothesis should be tested as to whether raising the haemoglobin level in patients with GCTs undergoing chemotherapy might improve the treatment outcome.

The presented data also allow for some conclusions regarding the mechanisms involved in the relative drug resistance induced by hypoxia. The impact of hypoxia on chemosensitivity depended strongly on the cell line. The least effect was evident in NT2, the only cells that kept proliferating under hypoxic conditions. The two remaining cell lines showed a far more pronounced relative drug resistance in hypoxia. These findings allow for two different interpretations: NT2 could be less sensitive for the effect of hypoxia in general, that is, hypoxia-induced effects are less pronounced. Alternatively, despite similar changes in hypoxia-induced gene expression overall, only the effect on cell proliferation differs between the cells. The latter interpretation would point to the effect on proliferation as the main factor determining the relative effect of hypoxia on drug sensitivity. However, there was no strict correlation between cell proliferation and cytotoxic effect indicating relevant influences of factors other than proliferation.

The hypoxia induced relative resistance to cytotoxic agents depended only partly on the specific substance. Despite the different modes of action – for example, for gemcitabine introduction of single-strand DNA breaks, and for etoposide and irinotecan topoisomerase inhibition – the relative increase in resistance to these drugs during hypoxia was similar. So far, the potential relevant resistance mechanisms for some of the drugs with similar behaviour under hypoxic and normoxic conditions have been considered to be nonoverlapping. Of the substances tested, only etoposide is transported out of the cells by P-glycoprotein (P-gp). Paclitaxel – a stabiliser of *β*-microtubulin polymerisation disrupting the formation of the normal mitotic spindles and thereby blocking mitosis (Horwitz, 1992) – is independent of P-gp (Lautier *et al*, 1996). Therefore, a HIF-1*α*-mediated induction of P-gp under hypoxic conditions – as recently proposed by Wartenberg *et al* (2003) – can be ruled out as a dominating resistance mechanism in hypoxia in our setting. Bleomycin causes DNA breaks through direct binding to DNA. This process depends on oxygen and produces reactive oxidative species (ROS), which may also play a role in the toxicity of bleomycin (Sikic, 1986). P53 does not seem to play an essential role in drug resistance under hypoxic conditions in the models chosen here, as NCCIT cells express mutant p53, and NT2 and 2102 EP express wild-type p53 (Burger *et al*, 1997). The broad spectrum of substances with unrelated modes of action and potential means of resistance suggests that rather universally active mechanisms or coactivation of several pathways confer resistance under hypoxic condition. Kinoshita *et al* (2001) reported that cancer cells might obtain resistance to apoptosis once they have survived hypoxia. The underlying mechanism remains elusive so far. Other investigators have also suggested that tumour cells acquire antiapoptotic features and will be selected by hypoxia (Kim *et al*, 1997).

In summary, this extensive *in vitro* study using several cytotoxic drugs in three TGC tumour cell lines shows the importance of normoxic conditions regarding treatment sensitivity in this tumour model for all chemotherapy agents investigated.
